# Does the Dental Profession Progress?

**Published:** 1863-12

**Authors:** 


### DOES THE DENTAL PROFESSION PROGRESS?

By T.

Frequently, when it is suggested that it is the duty of every one to do something for the advancement of his profession, and that every one should write, we are met with the question, "What shall I write upon? I can think of nothing new, and all the old subjects are worn threadbare." And hence the question—Have we ceased to progress? is the dental profession at a stand-still? has it arrived at the point where, either from the difficulties encountered or from the perfection attained, no further progress is possible? Certainly not the latter; for no one will pretend to assume that he has attained perfection, in any particular of our profession. Or is it that there are unconquerable difficulties in the way? Patience, labor and perseverance will overcome almost all obstacles. The fact is, such querists are not usually first-class students. They are content to plod along in the channels dug out by others; they do not wish to take hold of untried ways and processes; they are content, if somebody else will open up the mine, to work it, and especially if there is a rich *yield*.

The studious, inquiring, and persevering, will always have more to think and study upon, than he can transfer to paper. Such an one will always write some, perhaps much; and it will command attention too.

There is no surer indication of a retrograde movement—"advance backward"—than for one to say—I see nothing new or interesting in our profession.

Now we should like much to have the time and ability to answer this question at length; but for the present we must be content with a few remarks upon the subject in a general aspect.

And to this question we would answer, unhesitatingly, there *is* progress, rapid, great and permanent, in the dental profession. And we will not make any special effort to prove it either; for we regard it as most palpable to every one who has his eyes open.

Perhaps it may not be amiss to examine briefly some particulars in which progress is manifest.

In order that there should be progress, it is not necessary that some great and startling development should transpire. The greatest changes in the world, both physical and spiritual, are carried on silently. Occasionally the tornado comes with its whirl and dash, throwing all nature into the intensest agitation; and now and then, at long intervals, the windows of heaven are thrown wide open, and its watery treasure dashed in unmeasured quantity upon the earth, breaking up and washing away its surface; but these are not the usual methods of operation. Aerial circulation is, for the most part, imperceptible, except by close observation. The great proportion of rain comes to the earth in gentle showers, or the quiet mist, or the imperceptible dews: these clothe the earth in living green, and unlock its stores, and cause its treasures to come forth in unmeasured quantities.

Now, not quite so silent nor so imperceptible as this, has been the development and progress of our profession. It has risen, in comparatively a few years, from a system of quackery (there have always been noble exceptions), or, at best, a mere trade, to a recognized branch of the medical profession.

The progress of the dental profession has been characterized by two features, to-wit: 1st, by the great, sudden and rapid evolution of some available principle or particular of practice; and, 2d, by the great and rapid diffusion of knowledge among the members of the profession as a whole, and the practical attainments that necessarily accompany an increase of knowledge.

The latter thought is the more pleasing one to contemplate just now. Ignorance has been the great bane of our profession. Now I hope no one will make a personal application of this! and become offended; of course we do not expect to find the person to whom this applies, nor do we desire to do so; but, like the man who went hunting, when he heard a noise in the bush, blazed away without seeing his game; so we have heard a *noise*, and the game is certainly *there*, and shoot we must.

It is apparent to every one, that there is a far greater desire manifested in the profession, for the attainment of knowledge now, than formerly; and almost every legitimate desire finds means of gratification. So it is, at least, with this.

The fact of associated effort becoming so general, is an evidence of an increasing desire for knowledge. The time when dentists feared to confer with one another, lest ignorance would be exposed, or the world gain something by such conference, has passed away. Men now no longer fear or refuse to communicate with one another; they have learned that much is to be gained thereby.

There is much progress being made in the manner of performing operations, and, consequently, in their efficacy. There is a very apparent improvement in the character of operations upon the natural teeth, compared with those of a few years ago; this is dependent, to some extent, upon the improvement in instruments, appliances and materials; but all this would not avail much, without the ability to properly employ them.

This progress is not confined to manipulative ability; for it is, of necessity, based upon attainments in principles.

One of the most cheering evidences of progress in our profession is found in the fact, that there is an earnest and increasing searching and investigating of foundation-principles, especially in physiology and pathology. The desire to understand, control, modify and remedy disease, is now a very general one in the profession. A very little while ago, such an idea was wholly ignored, with a few exceptions.

Formerly, the teeth were operated upon, by the great mass of dentists, as though they were inorganic bodies, non-vital,—saying nothing of their susceptibility to pathological conditions. That day has passed away; and he who now occupies that position is scarcely, if at all, recognized as a member of the dental profession; neither should he be.

We are not now considering the progress that is being made by those who have been recognized as being in an advance position, but it is the movement of the main body, that we feel most interested in just now; and when that is accomplished, then let a united effort be put forth for the attainment of the highest point of excellence possible. We fully recognize the fact, however, that all can not travel with equal rapidity, Neither would we be understood as desiring to arrest the progress of the van-guard, but that they shall aid in executing the work we have suggested here; this would not retard them in their onward course, but give them increased strength, vigor and zeal for their work.

The fact that the people demand better operations than formerly, is an indication of progress.

